# Fixational eye movements during viewing of dynamic natural scenes

**DOI:** 10.3389/fpsyg.2013.00797

**Published:** 2013-10-29

**Authors:** James A. Roberts, Guy Wallis, Michael Breakspear

**Affiliations:** ^1^Systems Neuroscience Group, QIMR Berghofer Medical Research InstituteHerston, QLD, Australia; ^2^School of Human Movement Studies, University of QueenslandSt. Lucia, QLD, Australia; ^3^Queensland Brain Institute, University of QueenslandSt. Lucia, QLD, Australia; ^4^Royal Brisbane and Women's HospitalBrisbane, QLD, Australia; ^5^School of Psychiatry, The Black Dog Institute, University of New South WalesSydney, NSW, Australia

**Keywords:** fixational eye movement, drift, natural vision, random walk, anomalous diffusion

## Abstract

Even during periods of fixation our eyes undergo small amplitude movements. These movements are thought to be essential to the visual system because neural responses rapidly fade when images are stabilized on the retina. The considerable recent interest in fixational eye movements (FEMs) has thus far concentrated on idealized experimental conditions with artificial stimuli and restrained head movements, which are not necessarily a suitable model for natural vision. Natural dynamic stimuli, such as movies, offer the potential to move beyond restrictive experimental settings to probe the visual system with greater ecological validity. Here, we study FEMs recorded in humans during the unconstrained viewing of a dynamic and realistic visual environment, revealing that drift trajectories exhibit the properties of a random walk with memory. Drifts are correlated at short time scales such that the gaze position diverges from the initial fixation more quickly than would be expected for an uncorrelated random walk. We propose a simple model based on the premise that the eye tends to avoid retracing its recent steps to prevent photoreceptor adaptation. The model reproduces key features of the observed dynamics and enables estimation of parameters from data. Our findings show that FEM correlations thought to prevent perceptual fading exist even in highly dynamic real-world conditions.

## 1. Introduction

Fixational eye movements (FEMs) have seen considerable recent interest for their roles in perception and oculomotor control (Martinez-Conde et al., 2004; Engbert, 2006; Rolfs, 2009; Martinez-Conde et al., 2013). Their cause and functional significance remain poorly understood, but they appear to be much more than a noisy inconvenience—neural responses fade rapidly in their absence (Coppola and Purves, 1996).

Eye movements are broadly categorized into fixations (where the eyes are relatively still), pursuits (eyes smoothly tracking a moving target), and saccades (rapid movements between fixations). Fixations and pursuits are particularly important as it is during these periods that visual information is extracted from the world. Three types of FEM perturb this extraction: drift, tremor, and microsaccades (Martinez-Conde et al., 2009; Rolfs, 2009). Drifts are slow eye movements that follow apparently random trajectories and carry retinal images across several photoreceptors during typical fixations. Tremor has a broadband frequency spectrum (30–120 Hz, peak ~80 Hz), slightly perturbing drifts by <1 photoreceptor width (McCamy et al., 2013). Microsaccades are similar to regular saccades but have smaller amplitude similar to the displacements caused by drifts (Rolfs, 2009).

Fixational eye movements have been widely assumed to be a random uncorrelated process similar to Brownian motion (Pitkow et al., 2007; Burak et al., 2010; Kuang et al., 2012). Recent studies have shown that drift trajectories during fixation tasks contain non-trivial temporal correlations (Engbert and Kliegl, 2004; Mergenthaler and Engbert, 2007). On short time scales, correlations cause gaze to wander faster than expected for a normal diffusive process. Such processes are termed *superdiffusive* (Metzler and Klafter, 2000) or *persistent* (Codling et al., 2008). On longer time scales, trajectories are anticorrelated (or *antipersistent*), tending to wander slower than normal diffusion, consistent with a *subdiffusive* process (Engbert and Kliegl, 2004; Mergenthaler and Engbert, 2007).

Short-time superdiffusion is thought to refresh retinal images, with longer-time subdiffusion keeping gaze near fixation targets (Engbert and Kliegl, 2004). However, it is unknown whether these correlations exist in natural vision—superdiffusion may be unnecessary in rich dynamic environments, and subdiffusion could be an artifact of lengthy forced fixation. Natural scenes in films are inherently dynamic and arguably a better model of natural environments than typical fixation-task stimuli, and thus are an increasingly important paradigm in neuroscience (Felsen and Dan, 2005). Well-directed films are also particularly engaging for viewers (Hasson et al., 2004, 2010), sustaining attention during lengthy data acquisitions. Drifts and microsaccades have not (to our knowledge) been previously studied together during dynamic natural scene viewing.

Here, we characterize FEMs in natural vision by recording eye movements during film viewing. We show that gaze trajectories are well-described by a correlated random walk, with scaling properties similar to those in forced fixations. Moreover, we propose a model that generates the observed short-time correlations via an imprecise memory of recently visited gaze locations, and show that it captures the superdiffusive nature of FEMs at short time scales (≲100 ms). Finally, we show as a proof of principle that the model can be inverted to estimate its parameters from data.

## 2. Materials and methods

### 2.1. Data acquisition

Eight healthy subjects (3 female, mean age 25.9 years, range 22–28) viewed the Alfred Hitchcock (1948) film *Rope* (duration 77 min) on an LCD monitor, with the audio stream played through headphones. *Rope* is notable for having only ten director's cuts (five of which are masked) and is thus an almost completely continuous audiovisual stream. Subjects provided informed consent and the study protocols were approved by ethics boards of the Queensland Institute of Medical Research and the University of Queensland in accordance with the Declaration of Helsinki. Analysis was restricted to the 75 min segment beginning at 1 min 53 s spanning the time between the opening and closing credits. The video was presented in the center of the screen (surrounded by a black background), subtending horizontal and vertical angles of 20° and 16°, respectively (screen resolution ~35 px/degree). Eye movements were recorded with an SR Research EyeLink II eye tracker that allowed the head to move freely, sampling at *f*_*s*_ = 500 Hz using infrared cameras to record pupil position and correct for head motion. Subjects optionally took breaks every 15 min, with re-calibration on resuming the recording.

Fixations were identified as the intervals between saccades, which were detected by the eye tracker using velocity and acceleration thresholding. A saccade was deemed to be in progress if eye-movement velocity exceeded 30°/s and accelerations were in excess of 8000°/ s^2^. A saccade was said to start if these criteria were met for over more than two sampling periods, and continued as long as the criteria were met again within the next 20 ms. The eye tracker detected blinks as corresponding to a loss of pupil visibility, which is accompanied by spurious saccades immediately before and after the detected blink; both these saccades and the following 50 ms of gaze samples were discarded as part of the one blink. Blinks closer than 100 ms were merged and those gaze points in between discarded. Saccade overshoots and saccade onsets were deleted from the fixations by discarding 6 ms at the start and 4 ms at the end of each fixation, respectively. Both eyes were recorded, but only the left eye's data was used in all subsequent analysis; results for the right eye were similar.

The PDFs of step lengths and turn angles were estimated from histograms of these quantities aggregated across all fixations in each subject. Because the eye tracker reports gaze coordinates to the nearest 0.1 px, histograms of the raw steps are sharply discretized, particularly for the turn angles. Thus we added small amplitude noise to all gaze points uniformly distributed between −0.05 px and 0.05 px to smear the discretization imposed by finite measurement precision. This only affects the step length distribution for very short steps of ≲0.1 px so this perturbation is negligible for most steps but sufficient to yield smooth turn angle PDFs.

## 3. Results

Table 1 summarizes fixation counts and durations for each subject. Note that Subject 1's recording was limited to the first 50 min of the film. Subjects made 1.7–2.7 fixations per second, resulting in fixation durations (mean across all subjects 424 ms, median 290 ms) an order of magnitude or more shorter than used in typical fixation tasks (Engbert and Kliegl, 2004; Mergenthaler and Engbert, 2007).

**Table 1:** **Summary of subject fixation statistics**.

**Subject**	***N***	**Mean dur (ms)**	***H***_1_	***H*_peak_**	**Δ*t*_peak_ (ms)**
1	4848^*^	553	1.53	1.66	6
2	10230	404	1.34	1.61	14
3	11932	334	1.50	1.58	6
4	10106	388	1.36	1.52	14
5	8870	461	1.42	1.51	10
6	9009	421	1.37	1.53	14
7	9219	415	1.37	1.44	14
8	7896	524	1.36	1.40	6

* Denotes recording aborted after 50 min.

### 3.1. Properties of fixational eye movements

**Figure 1:**
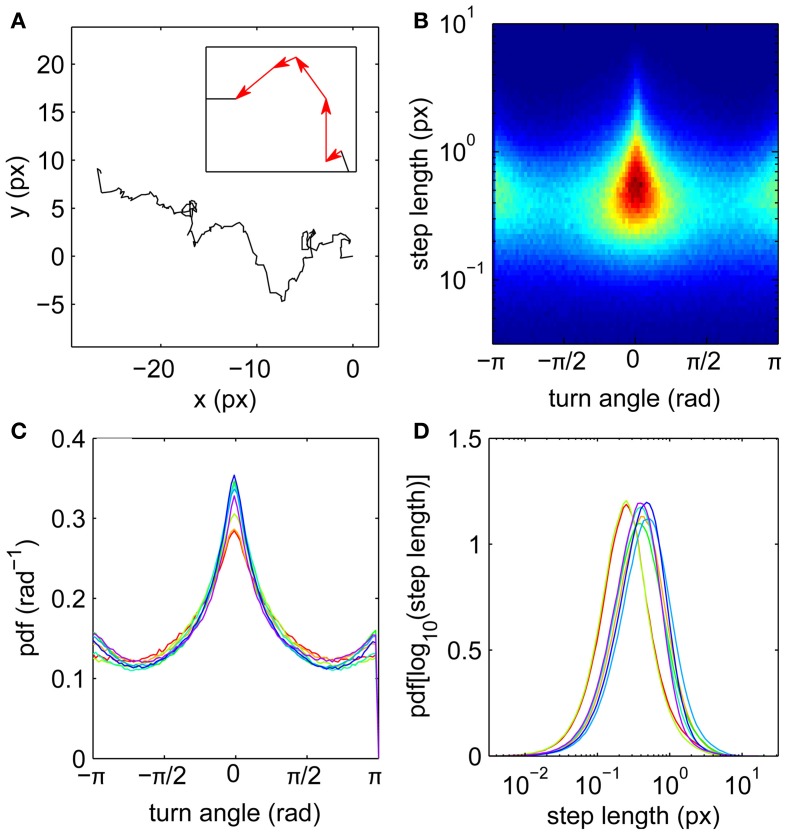
**Fixational eye movements and distributions of steps. (A)** Example fixation gaze trajectory, with inset showing four steps as vectors joining gaze points. **(B)** Joint step length and turn angle distribution for one subject. **(C)** Turn angle distributions for eight subjects. **(D)** Step length distributions (log transformed) for eight subjects. Subjects 1–8 colored in order red, yellow, light green, green, cyan, light blue, blue, purple.

Motivated by the apparently random gaze trajectories observed during fixations, we analyze each trajectory as a random walk composed of a series of steps joining the measured gaze coordinates (Figure 1A). The steps are equally spaced in time because of the digital acquisition, each described by its length (which is proportional to the instantaneous velocity) and turn angle θ measured relative to the previous step. The steps can thus be treated as vectors (see inset). Step distributions (Figures 1B,C) reveal anisotropy in the random walk trajectories. Forward steps (θ ≈ 0) are both more numerous and longer than those in other directions, as indicated by the central triangular zone in the single-subject step distribution shown in Figure 1B. Thus the eye tends to maintain its direction during drifts, consistent with short-time persistence in the trajectories (Engbert and Kliegl, 2004). This does not rule out backward steps; on the contrary, drifts in the reverse direction are more probable than left or right turns (Figure 1C).

**Figure 2:**
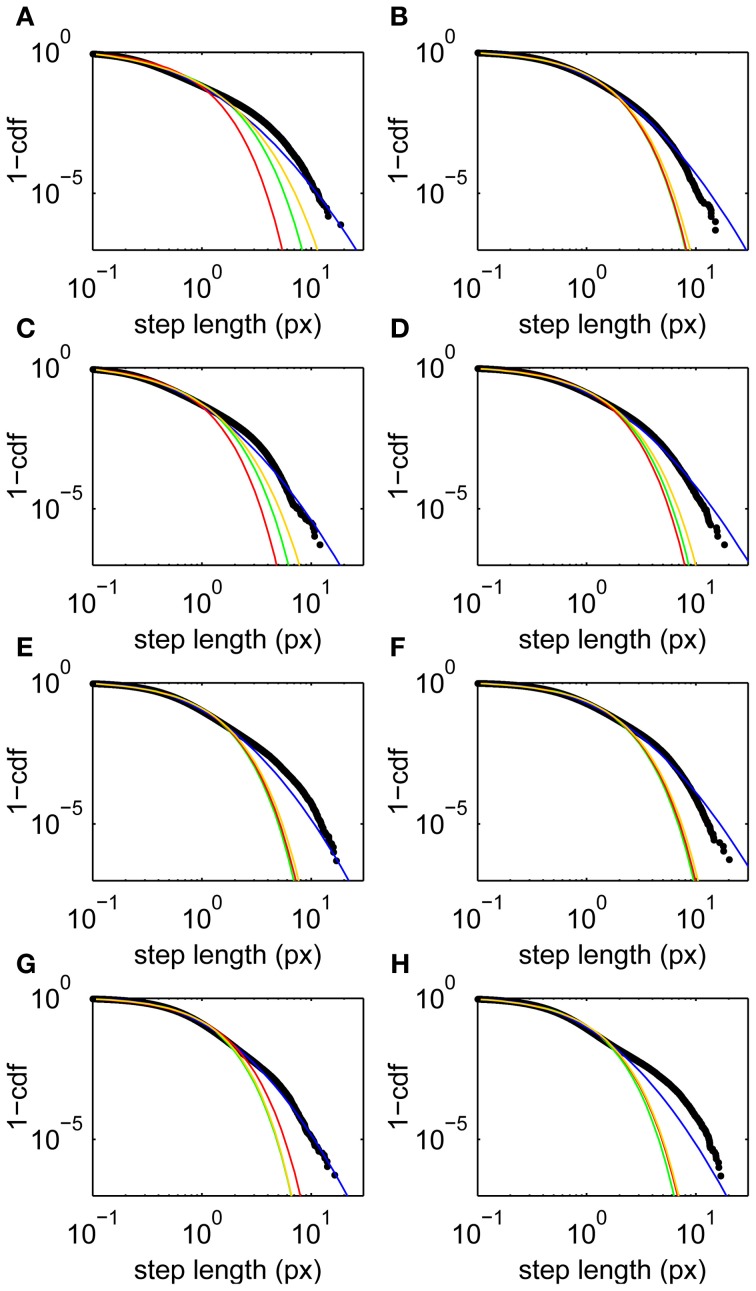
**Step length upper cumulative distribution functions and fits for all subjects**. Data (black) and fits to lognormal (blue), Weibull (yellow), power law with exponential cutoff (green), and exponential (red) distributions. **(A–H)** Subjects 1–8, respectively. Fits are maximum likelihood estimates to the tail above step lengths of 0.1 px using the methods of Clauset et al. (2009).

Step lengths are unimodally distributed (Figure 1D), approximately following a lognormal distribution (Figure 2). This long-tailed distribution implies that long steps are more frequent than expected for a normally-distributed variable, but there is no evidence for a second mode of outliers that would suggest the presence of microsaccades (Engbert and Mergenthaler, 2006). Such a mode would be expected if a significant number of fixations were punctuated by a large number of flighty gaze paths an order of magnitude faster than the drifts.

As a more sensitive test for microsaccades we apply the detection method of Engbert and Mergenthaler (2006), restricting attention to binocular microsaccades and discarding as false positive saccade overshoots any within a 30 ms interval centered on (normal) saccades, similar to a study of free-viewing data (Mergenthaler and Engbert, 2010). The method protects against noise by comparing the detected microsaccade rate to that of amplitude-adjusted Fourier-transformed surrogate data that preserves velocity distributions—the excess microsaccade rate in the data over the surrogates thus estimates the true microsaccade rate. Using this more sensitive method, we find 0.77 microsaccades per second (range 0.5–1.1 s^−1^) averaged across all subjects, slightly lower but broadly consistent with the rate 1.0±0.4 s^−1^ reported during free-viewing of static natural stimuli (Mergenthaler and Engbert, 2010). Given typical fixation lengths of ~400 ms, a rate below 1 s^−1^ implies that a majority of fixations do not contain microsaccades.

Moving beyond the statistics of individual steps, correlations between steps modify the rate at which gaze moves away from an initial point. The mean square displacement 〈Δ*x*^2^〉 after a time interval Δ*t* scales as 〈Δ*x*^2^〉 ∝ Δ*t^H^* with scaling exponent *H* = 1 for normal diffusion, *H* > 1 for superdiffusion or a persistent random walk, and *H* < 1 for subdiffusion or an antipersistent random walk. The estimator *D*^2^ for 〈Δ*x*^2^〉 is given by

(1) $$D^{2}(m)=\frac{1}{N-m}\sum_{i=1}^{N-m}\|x_{i\;+\;m}-x_{i}{\|}^{2},$$

where *m* indexes time within fixation trajectories **x**_*i*_ =(*x*_*i*_, *y*_*i*_) (so in physical units Δ*t* = *m*/*f*_*s*_) and *N* is the number of points in the time series (Engbert and Kliegl, 2004).

**Figure 3:**
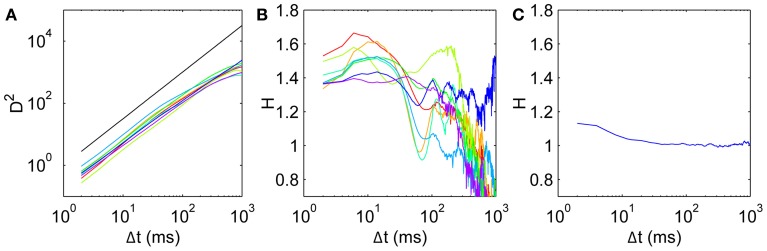
**Mean squared displacement and scaling exponent for all subjects. (A)** *D*^2^, with a reference line (black) with slope *H* = 1.5. **(B)** Scaling exponent *H* for mean square displacement given by the gradients of the *D*^2^ curves. Subjects 1–8 colored in order red, yellow, light green, green, cyan, light blue, blue, purple. **(C)** Scaling exponent for a surrogate walk with uncorrelated steps drawn from the empirical anisotropic step distribution in Figure 1C.

For all subjects, the mean square displacement *D*^2^ (Figure 3A) grows more quickly at short time scales than expected for uncorrelated dynamics, with *D*^2^ ~ Δ*t*^1.5^ consistent with superdiffusive dynamics, before tapering off at long times. This behavior is consistent across all subjects, although there is some variation in the precise Δ*t* dependence. To quantify this, we calculate the scaling exponent *H* given by the log-log slope of *D*^2^ vs. Δ*t*, shown for all subjects in Figure 3B. Common to all subjects, the initial scaling exponent *H*_1_ (the *D*^2^ log-log slope between Δ*t* = 2 ms and Δ*t* = 4 ms) is in the superdiffusive regime with *H*_1_ = 1.41 ± 0.07 (mean ± SD). The *H* curves all remain in the superdiffusive regime for Δ*t* ≲ 30 ms, rising to a peak of *H*_peak_ = 1.53 ± 0.09 at corresponding time lag Δ*t*_peak_ = 11 ± 4 ms. The peak is particularly distinct in seven subjects (1–7), while the remaining subject (8, purple) exhibits a flatter *H* with a small peak at Δ*t* = 6 ms. The three parameters *H*_1_, *H*_peak_, and Δ*t*_peak_ are thus a useful characterization of the short-time scaling behavior. Single-subject values are given in Table 1.

At longer time scales, the *H* curves dip to a minimum at Δ*t* ≈ 60–80 ms in six subjects (1,2,4–7), with two subjects's exponents dipping into the *H* < 1 subdiffusive regime. Five subjects exhibit a shallower local minimum *H*, remaining in the superdiffusive regime. At longer time scales again, all the *H* curves increase again to peak at Δ*t* ≈ 100–200 ms, and become diffusive or subdiffusive on time scales of Δ*t* ≳ 400 ms on the order of the entire fixation length. Subject 7's data (blue) is in the superdiffusive regime even at these long time scales, and exhibits oscillations.

Our analysis of mean squared displacement is motivated by previous studies (Engbert and Kliegl, 2004; Mergenthaler and Engbert, 2007) and has the benefit that the results are immediately relatable to gaze position. Equivalently we could have analyzed spectra in the Fourier domain—scaling exponents and spectral exponents are closely related (Mandelbrot and Van Ness, 1968). Power spectra of eye position during fixation are known to reveal approximately 1/*f*^2^ spectra at low frequencies (Eizenman et al., 1985; Kuang et al., 2012), and deviations from this functional form indicate the presence of processes other than Brownian motion, and indeed this is observed at high frequencies (i.e., short time scales), consistent with our time-domain findings.

It is important to test whether the observed correlations can be explained solely by the observed anisotropy in the turn angle distribution. A purely uncorrelated 2-D random walk would have uniform turn angle distribution, whereas anisotropy imposes short-time correlations even when the individual steps are drawn independently. Thus we construct a surrogate walk with steps randomly chosen from the empirical distribution in Figure 1D and estimate the scaling exponent as above. The resulting surrogate walk is initially weakly persistent with *H* = 1.13 (Figure 3C). This exponent decays to *H* < 1.05 for Δ*t* ≳ 10 ms, consistent with normal diffusion and strongly inconsistent with the corresponding scaling behavior observed empirically (Figure 3B). This can also be shown by shuffling the order of the step vectors and reconstituting them to generate a surrogate walk that preserves step distribution by construction (Engbert and Kliegl, 2004). Thus the random walk must be correlated beyond the extent imposed by anisotropy in its movements.

**Figure 4:**
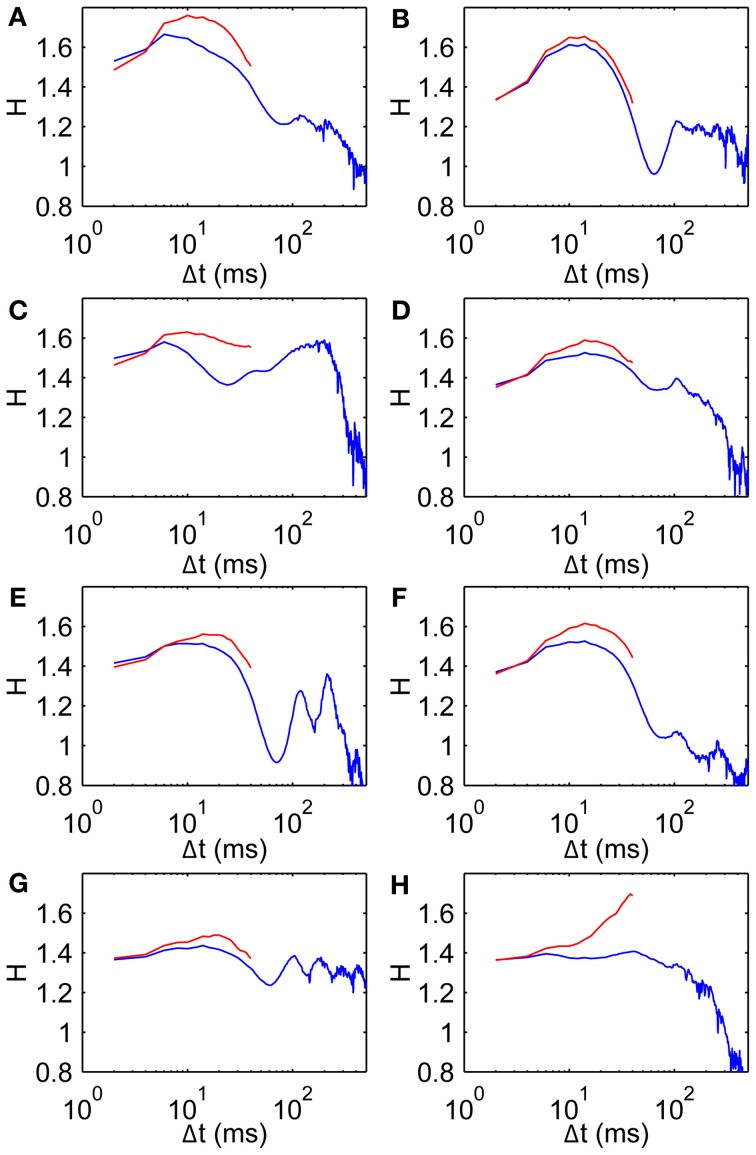
**Scaling exponents for all subjects when restricted to middle 50 ms of each fixation. (A–H)** Subjects 1–8, respectively, showing scaling exponent *H* data (blue, same as Figure 3A) and for the middle 50 ms of each fixation (red).

It is also conceivable that drift correlations could arise from muscle tension remaining after a saccade. To test this we calculate *H* using only the middle 50 ms of each fixation, thereby keeping well away from nearby saccades by restricting the analysis to a central time window of each individual FEM epoch. Scaling exponents restricted in this way are broadly similar to those using the original wider range (Figure 4). In particular these data on the middle 50 ms of each fixation show the same basic trend of *H* beginning in the superdiffusive regime, increasing over short time scales, then falling over medium time scales. Note that the rightmost points on the red curves are averages over fewer observations than the corresponding points on the blue curves (hence subject 8's sharp peak is likely a spurious effect of limited observations). Interestingly, in all cases the middle 50 ms is *more* superdiffusive than the uncut fixations—the superdiffusive behavior is thus not an artifact of recent/imminent saccades.

**Figure 5:**
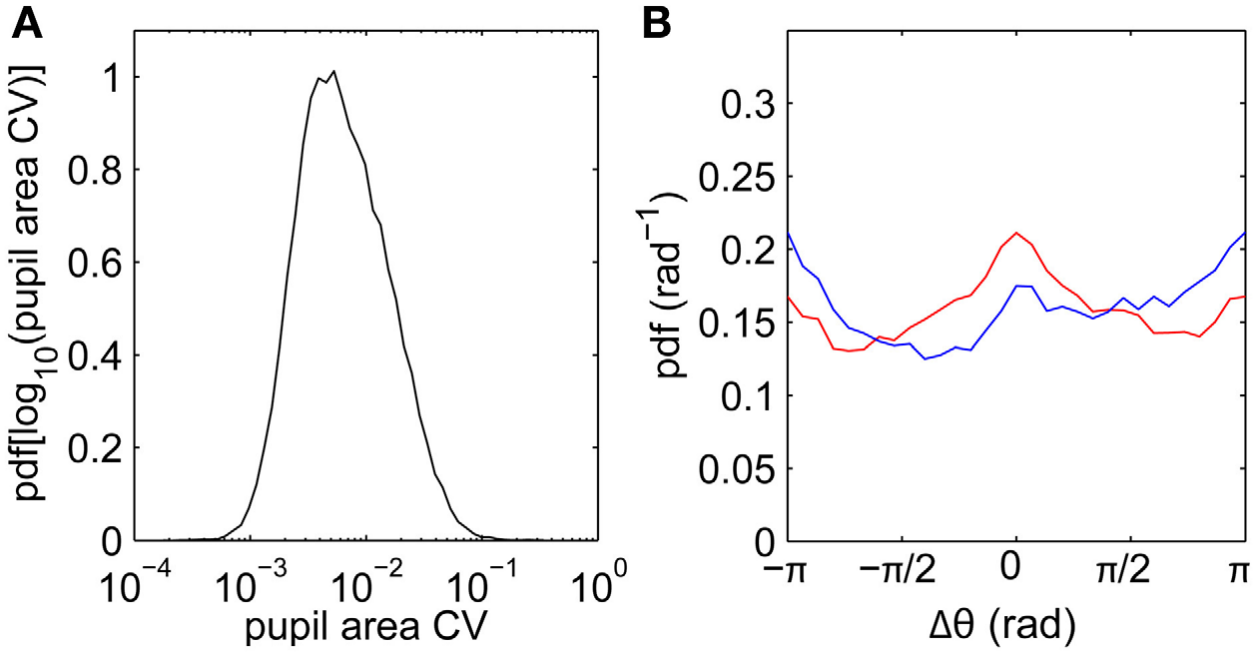
**Pupil area fluctuations and relationships between fixation and saccade directions. (A)** Distribution of pupil area coefficient of variability (CV; SD/mean) calculated for each fixation, data pooled across all subjects. **(B)** Difference Δθ between fixation drift angle (direction of vector joining fixation start to fixation end) and previous saccade angle (blue), and between fixation angle and next saccade angle (red).

Another potential contribution to drifts measured using video-based eye trackers is cross-talk between pupil size and position following sudden luminance changes (Kimmel et al., 2012). To quantify the magnitude of pupil size fluctuations, we calculate the coefficient of variation (SD/mean) of pupil size across each fixation. The distribution of pupil size varied only slightly within each fixation (Figure 5A), with a median within-fixation change of 0.6% across all subjects. This is consistent with our film stimuli having relatively slowly-varying luminance. Pupil size fluctuations are thus unlikely to be a significant factor in determining the drift statistics.

Beyond turn angles, we can also characterize drifts by their overall directions relative to preceding and following saccades. Figure 5B shows the distribution of fixation angles (directions of vectors joining first and last points) relative to the preceding saccade angles (blue) and to the next saccade angles (red), pooling across all subjects. The main feature is that the drift direction distributions are relatively uniform, such that drifts are not strongly driven by the previous and next saccades. As a weaker effect, small peaks in the distributions suggest small biases in the directions parallel (Δθ = 0) and antiparallel (Δθ = ±π) to the neighboring saccades. Drifts are slightly biased against the previous saccade direction, consistent with the existence of slow corrective motions following saccades (Weber and Daroff, 1972). Drifts are slightly biased toward the next saccade direction, possibly reflecting “stick-slip” trajectories during pursuits.

**Figure 6:**
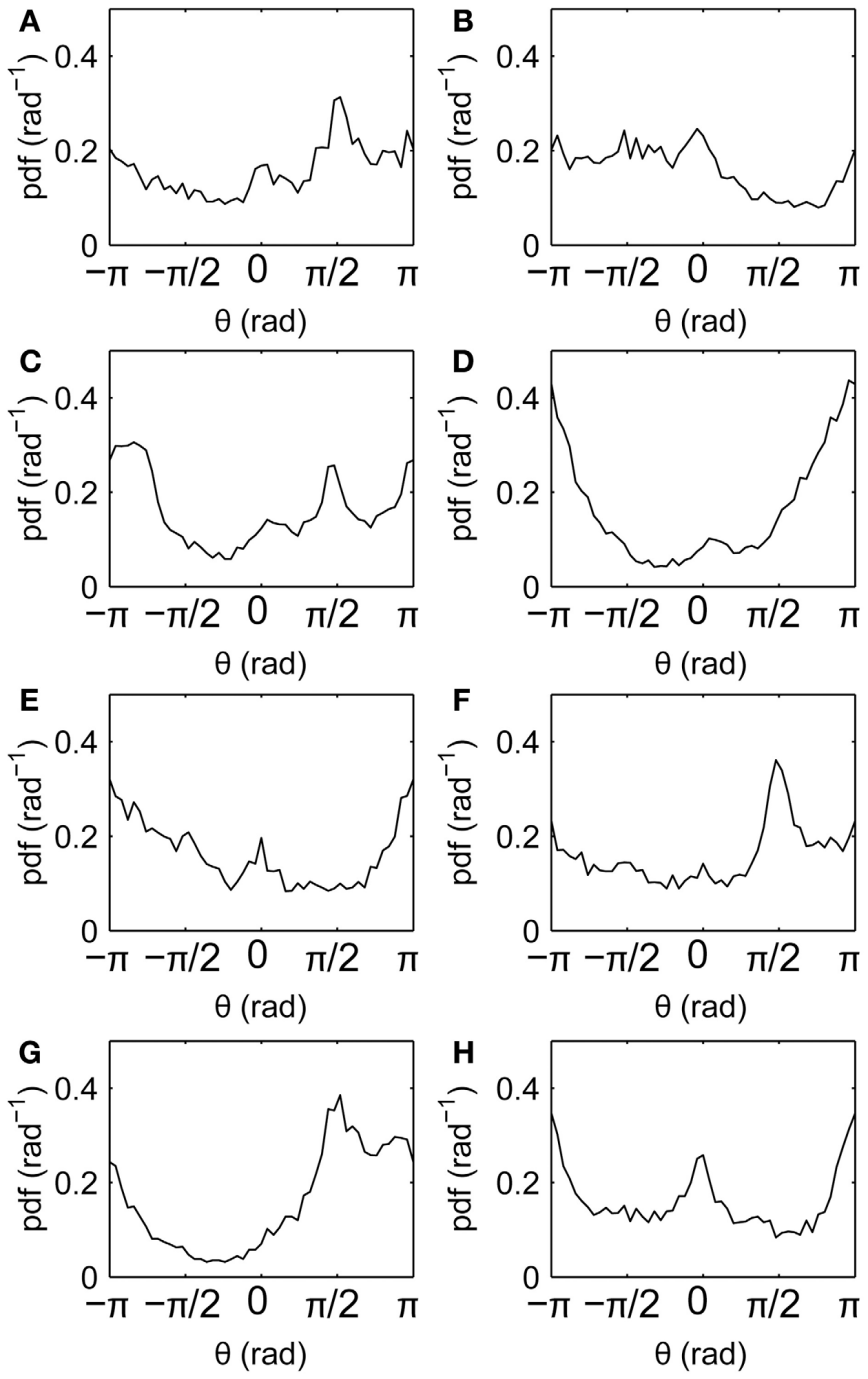
**Fixation drift directions relative to the screen. (A–H)** Subjects 1–8, respectively, showing distributions of fixation drift angles θ, where θ = 0, ±π denotes horizontal drifts and θ = ±π/2 denotes vertical drifts.

Drift directions measured relative to the screen reveal anisotropies (Figure 6), with drifts tending to follow the horizontal and vertical axes (θ = 0, ±π/2, ±π). This is similar to the well-known cardinal bias in saccade directions. Here, not all axes are favored in all individuals, suggesting that the bias is idiosyncratic rather than driven directly by the film.

### 3.2. Model of fixation drifts

The correlations dominating the observed short-time drift dynamics imply a memory of past gaze locations. In fact the scaling exponent *H* lies close to the exact exponent of *H* = 1.5 for a 2-D self-avoiding random walk (Madras and Slade, 1993). This is an idealized random walk on a lattice that is constrained to avoid all previously visited points. Self-avoiding walks have been widely used to model the physical configurations of polymers, where self-avoidance arises from the requirement that no two atoms occupy the same location. It is intuitively appealing to apply the self-avoiding random walk to FEMs because this mechanism would straightforwardly prevent adaptation to inputs. However, a problem with this approach is that the eye cannot be perfectly self avoiding; if it were it would rapidly become trapped with no possible steps remaining. The lattice restriction is also unrealistic given that the eye can turn through any angle, rather than just a small number of discrete choices.

**Figure 7:**
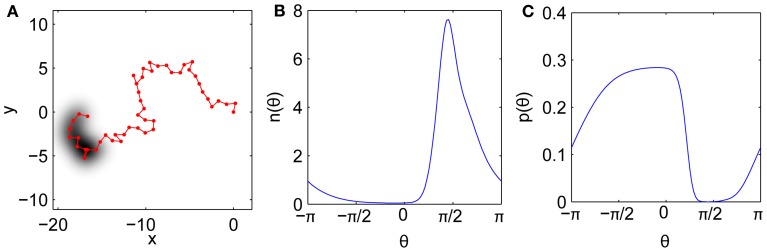
**Model mechanism. (A)** Example fixational gaze trajectory with imprecise history representation shaded. **(B)** History angular density *n*(θ) as measured from the current point. **(C)** Resulting angular distribution *p*(θ) for the next step after penalizing the history.

Here we construct an approximately-self-avoiding random walk that overcomes these limitations. The model's key ingredient is a brief, imprecise memory of recently visited points, which biases the step turn angles to avoid retracing this history. The bias is imposed by choosing each turn angle from a continuous distribution weighted by the density of recent gaze history in each direction. Thus the model tends to avoid directions that have been well traversed, but not as strictly as the idealized self-avoiding walk. Modified self-avoiding walks on the lattice that do not trap themselves have also been developed for modeling polymer growth (Amit et al., 1983; Kremer and Lyklema, 1985). Our simple model is illustrated in Figure 7. An example trajectory is shown in Figure 7A. Past locations are remembered imprecisely, with each recent gaze point Gaussian-blurred in space, consistent with the fact that the visual system has only finite spatial resolution and is noise-limited at the smallest scales. Figure 7B shows the corresponding angular density *n*(θ) of the history as “viewed” from the current gaze location. Directions with high *n*(θ) are penalized relative to directions that have not been visited recently, yielding the probability *p*(θ) for the next turn angle, shown in Figure 7C.

To describe the model in more detail, let **x**_*j*_ = (*x*_*j*_, *y*_*j*_) be a walk with steps indexed by *j*, and let **y**_*m*_ be the history that influences the dynamics (a subset of **x**_*j*_, or possibly the whole trajectory thus far). We seek the probability *p*(θ) that the next step **x**_*j*+*i*_ lies in the direction θ. For now we restrict our attention to walks with unit steps, but this could be relaxed to choose step lengths from the conditional distributions in Figure 1 (alternatively the step lengths could emerge from a more detailed model).

Assume that *p*(θ) ∝ *h*[*n*(θ)], where *n*(θ) is the number density of points in the direction θ, and *h*(*n*) is a function that penalizes directions with large *n*. We choose *h*(*n*) = exp(−α*n*), where α parameterizes the strength of the penalty (large α implies a strong penalty). An exponential penalty has also been used in a self-avoiding walk confined to a lattice (Amit et al., 1983). The important point is that *h*(*n*) is a decreasing function of *n*(θ), so that directions in which *n*(θ) is high will have a correspondingly small probability of being chosen for the next step. Hence the random walk will tend to avoid its history and move to relatively unexplored regions.

To calculate *n*(θ) we need to count the number of history points in each direction. For a continuous turn angle distribution (Δθ → 0), a perfect history represented as a set of points would result in only very precise angles being penalized (exactly the θ_*m*_, a set of measure zero), thereby yielding a uniform distribution and no memory effect. Instead, we note that the visual system has only finite spatial resolution, such that the representation of the history is blurred in space. We thus model the history as a sum of 2-D Gaussians given by

(2) $$n(x)=\sum_{m\;=\;1}^{\tau }\frac{1}{2\pi \sigma^{2}}\exp\text{​}\left[-\frac{{\left(x-x_{m}\right)}^{2}+{\left(y-y_{m}\right)}^{2}}{2\sigma^{2}}\right],$$

where σ is the width of the Gaussians and τ is the memory length (in time points, multiply by 2 ms for physical units); ∫ *n*(**x**)*d***x** = τ since the Gaussians are normalized. We assume that τ is finite and that the memory starts at the immediate previous point. Thus the step from **x**_*j*_ to **x**_*j*+1_ depends on points **y**_*m*_ = {**x**_*j*−1_, …, **x**_*j*−τ_}.

By converting Equation (2) to polar coordinates **x** = (*r*, θ) and **y**_*m*_ = (*r*_*m*_, θ_*m*_) centered on the current gaze location and integrating over *r*, the number of points contained in an infinitesimal range of angle *d*θ is

(3) $$n\left(\theta \right)=\frac{1}{2\pi }\sum_{m}e^{r_{m}^{2}/2\sigma^{2}}\left\{1+\sqrt{\pi }R_{m}(\theta )e^{R_{m}{\left(\theta \right)}^{2}}\text{erfc}\left[-R_{m}(\theta )\right]\right\}\text{​},\;\;$$

where $R_{m}(\theta )=r_{m}\cos(\theta -\theta_{m})/(\sigma \sqrt{2})$, and erfc is the complementary error function. Thus for the probability of choosing angle θ we have

(4) $$p\left(\theta \right)=\frac{\exp\text{​}\left[-\alpha n(\theta )\right]}{\int_{0}^{2\pi }\exp\text{​}\left[-\alpha n(\theta )\right]d\theta },$$

with *n*(θ) given by Equation (3).

For numerical simulation, walks are generated by iteratively drawing turn angles from the distribution *p*(θ), updated at each time step (Δ*t* = 2 ms) to incorporate the updated history. Simulation lengths are given in figure captions 8 and 9.

**Figure 8:**
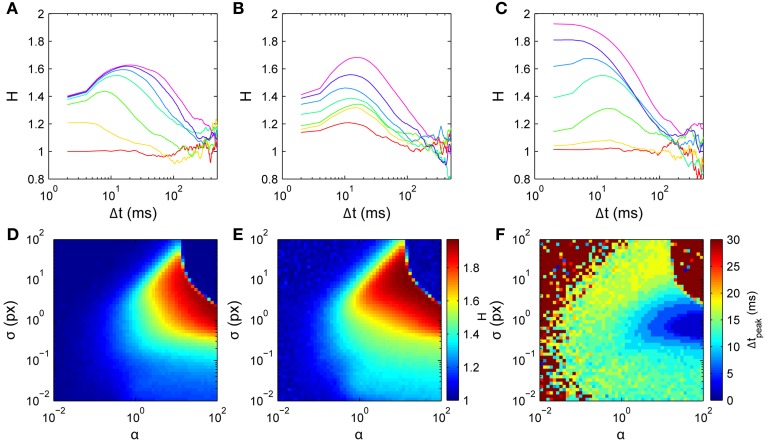
**Model scaling exponent parameter dependences**. Simulations used *D*^2^ curves averaged over 100 walks of 500 steps, around the nominal parameter set τ = 10 time points (20 ms), α = 1, and σ = 1 px. **(A)** Varying memory length τ; curves from bottom to top are for τ = 0, 2, 5, 10, 15, 20, 25 time points, respectively. **(B)** Varying memory precision σ; curves from bottom to top are for log_10_σ = −2, −1.6, −1.2, −0.8, −0.4, 0, 0.4, respectively. **(C)** Varying memory penalty strength α; curves from bottom to top are for log_10_α = −2, −4/3, −2/3, 0, 2/3, 4/3, 2, respectively. Bottom row: 2-D parameter space σ vs α with τ=10. **(D)** *H*_1_. **(E)** *H*_peak_. **(F)** Δ*t*_peak_.

**Figure 9:**
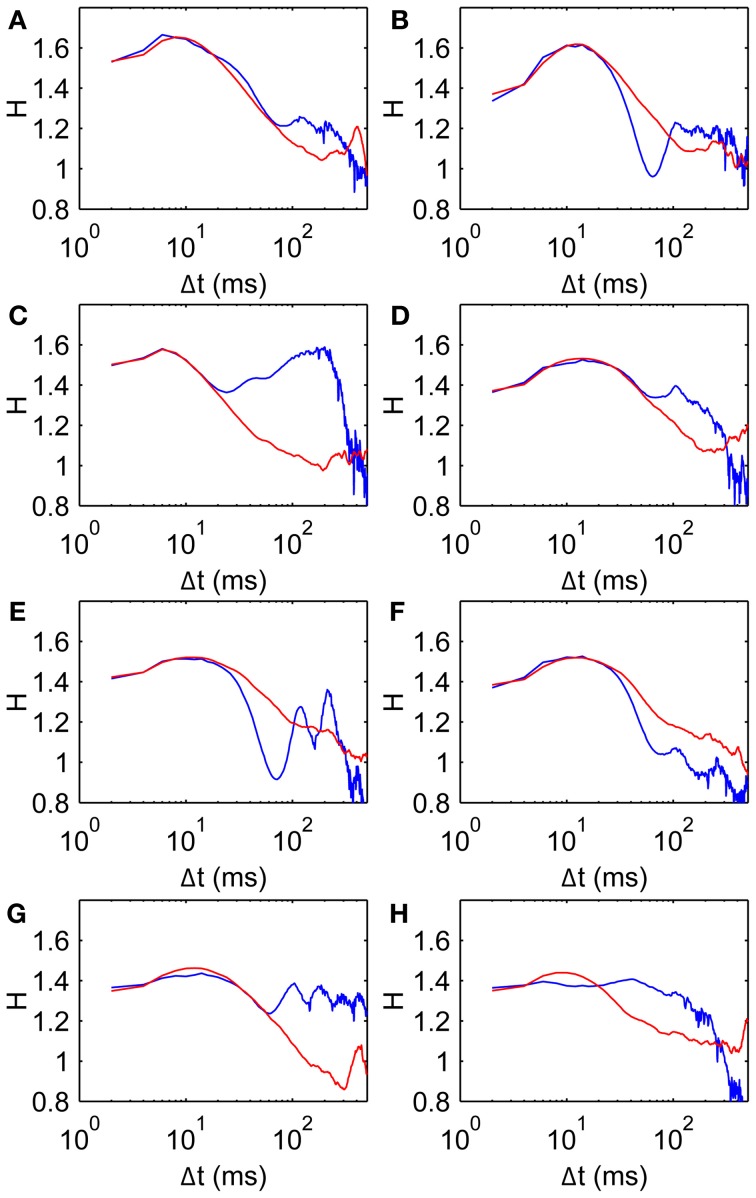
**Comparison data and model fits for all subjects**. Parameters given in Table 2. Model curves are obtained from average *D*^2^ curves of 100 walks of 500 steps. **(A–H)** Subjects 1–8, respectively, showing scaling exponent *H* data (blue, same as Figure 3A) and model fits (red).

### 3.3. Model dynamics

The model has only three parameters: the number of points in the memory τ (memory has finite length), the width of the blurred memory points σ (memory has finite precision), and the strength of the penalty for moving in previously-visited directions α. Parameters τ and σ in particular have a simple intuitive meaning in describing the temporal and spatial properties of the memory representation. We explore the model's behavior by varying these parameters about the nominal set τ = 20 ms (10 time points), α = 1, and σ = 1 px.

Memory length τ (Figure 8A) predominantly determines the dominant time scale in the scaling exponent *H*'s temporal profile. The overall shape agrees well with the data for Δ*t* ≲ ms, with *H* rising to a peak then falling to a value consistent with normal diffusion. For τ = 0 there is no memory and the dynamics are diffusive (*H* ≈ 1). For small τ > 0, increasing τ increases the initial *H* rapidly and yields a peak in *H* at non-zero Δ*t*. Increasing τ increases the height of this peak *H* and shifts it to the right, while *H* at small Δ*t* increases only weakly. Comparison with Figure 8B suggests that a short memory of τ = 5 points (10 ms) is consistent with the data.

Memory width σ (Figure 8B) predominantly determines the peak height in *H*'s temporal profile. For very small σ, the dynamics are diffusive because only very narrow angles are penalized. Increasing σ for small to moderate values yields increases in *H* (both for small Δ*t* and at the peak), with only a weak effect on the peak's Δ*t*. The effect of varying σ is thus approximately independent of the effect of changing τ. Further increasing σ to large values reduces *H*, leading again to normal diffusion with *H* ≈ 1 when the history is so broad that it extends even into the forward direction, penalizing all directions approximately evenly (upper regions of Figures 8D,E).

The history penalty strength α (Figure 8C) controls the degree of superdiffusivity in the dynamics. For small α the history only penalizes very heavily-visited directions, and hence the dynamics are approximately diffusive throughout. For large α, any visited directions are highly unlikely to be chosen. In particular, backward steps are unlikely, leading to approximately ballistic motion at short times (*H* ≈ 2), tending to normal diffusion at longer times. This is similar to a walk restricted to only a narrow range of turn angles, where many steps are needed before the dynamics can appear diffusive.

Thus the three parameters each act on distinct features of the *H* curves. Peak exponent *H*_peak_ is most strongly determined by σ, peak time *t*_peak_ is most strongly determined by τ, and initial exponent *H*_1_ is most strongly determined by α, which also smoothly modulates the overall dynamics between purely normal diffusion and strongly ballistic short-time behavior.

### 3.4. Parameter estimation from data

As a proof of principle, we use a simple model inversion approach to infer parameters from individual data sets. Our intention is to demonstrate how this simple model can be fitted to data, an important initial step before implementing a formal model inversion scheme. The method involves first numerically generating *H* curves across the 3-D parameter space, then using this set of curves as a lookup table to find the parameters that best fit the data-derived curves. Since τ is discrete, the space is adequately spanned by a series of 2-D slices in which σ and α vary at fixed τ. Moreover, since Δ*t*_peak_ increases with τ and our data all have Δ*t*_peak_ < 20 ms, only roughly twenty such slices are needed. One such slice is shown in Figures 8D–F. We sampled each 2-D slice with a 51 × 51 logarithmically-spaced grid in α and σ spanning the region explored in Figure 8. Best fits were chosen as those with the minimum sum of least-squared errors over the range of short time scales Δ*t* ≤ 20 ms.

Figure 9 shows *H* curves for both the data and the model using estimated parameter values listed in Table 2. The *H* data and fitted curves agree well for short time scales, with residual errors <5% for at least 20 ms in Δ*t* in all subjects. The good agreement between model and data extends beyond the fitted range in most subjects, up to at least 60 ms for three subjects (Panels A, D, and G). Three of the fits (Panels B, E, and F) agree well for the first 20–30 ms but do not have the same curvature; the dip in *H* appears to be too steep for the model. The dip in *H* is also made more pronounced by the data's trend toward *H* = 1 being interrupted by a second peak at Δ*t* = 100–200 ms.

**Table 2:** **Estimated model parameter values corresponding to scaling exponents of the data in Figure 9**.

**Subject**	**τ**	**σ**	**α**
1	5	1.74	2.51
2	7	19.1	5.25
3	3	3.02	4.37
4	12	0.692	1.00
5	13	0.229	7.59
6	12	0.575	1.20
7	10	0.132	11.0
8	8	0.110	83.2

## 4. Discussion

Fixational eye movements are an ever-present source of fluctuations in the visual input stream, continually perturbing retinal images during periods when information is extracted from the external environment. We have shown that FEM-induced fluctuations in the visual stream during natural vision are not simply additive measurement noise: they have a particular correlation structure, distinguishing them from simple uncorrelated processes such as Brownian motion. Our analysis yields insights into possible underlying mechanisms and links FEMs in natural vision to the broad class of anomalous diffusion phenomena occurring in diverse complex systems including vestibulo-oculomotor neurons, posture control, particle transport, bacterial motion, and animal foraging (Anastasio, 1994; Collins and De Luca, 1994; Viswanathan et al., 1999; Metzler and Klafter, 2000; Codling et al., 2008).

Studies of FEMs have mostly focused on data obtained with head restraints during forced fixation of static shapes, raising questions of the work's relevance to more ecologically-relevant activities (Collewijn and Kowler, 2008). Microsaccades have now been detected in several conditions with varying degrees of increased realism, including head-unrestrained fixation tasks (Martinez-Conde et al., 2006) and free-viewing (Otero-Millan et al., 2013), head-restrained fixation (Poletti and Rucci, 2010; Di Stasi et al., 2013) and free-viewing (Otero-Millan et al., 2008; Mergenthaler and Engbert, 2010; Otero-Millan et al., 2013) of static natural scenes, use of stimuli encompassing the entire visual field (Otero-Millan et al., 2013), fixation tasks with dynamic artificial stimuli (Laubrock et al., 2008), and in a dynamic simulated driving environment (Benedetto et al., 2011). Here, we presented dynamic natural scenes that more closely approximate real-world viewing conditions than do typical static stimuli. Our finding of correlated drift trajectories implies that these correlations are not merely responses to the constrained tasks and specialized featureless static stimuli used in many studies. Furthermore, because these correlations exist during viewing of dynamic stimuli, movement does not obviate the need for drifts. Motion is ubiquitous in the real world and thus many of the objects we fixate on will inevitably be moving targets, with trajectories that might be simple (cars driving past) or complicated (hand movements while someone talks)—visual input is ultimately a convolution of object motion with eye motion. The need to move beyond static images to dynamic and task-driven visual input is becoming widely accepted, particularly for modeling gaze allocation (Tatler et al., 2011) and gaze strategies that evolve over time (Wang et al., 2012). Film stimuli beneficially enable long recordings in response to an approximately smooth and continuous visual stream. While directed films are particularly engaging for viewers (Hasson et al., 2004, 2010), the presence of numerous cuts alters the gaze behavior relative to undirected videos (Dorr et al., 2010). Our use of *Rope* was motivated by it having relatively few cuts, thus being closer to natural conditions than typical films. Although static natural images could provide real-world scene statistics, building up robust statistics over a large ensemble of fixations would require presentation of many such images. This results in a long, discontinuous slideshow, characterized by recurring surprise then habituation, that is arguably less engaging and realistic than a single dynamic natural scene.

The observed drift scaling properties are broadly consistent with those found during fixation tasks (Engbert and Kliegl, 2004; Mergenthaler and Engbert, 2007). This includes the subdiffusive confining effect at medium-length time scales, implying that subdiffusion is not entirely due to the deliberate effort of lengthy precise fixation on a target. Nor do our findings attribute subdiffusion to corrective microsaccades, in agreement with previous findings (Mergenthaler and Engbert, 2007). We found no evidence for a distinct high-velocity component in the gaze step distributions and only a relatively low microsaccade rate consistent with free-viewing of static images (Mergenthaler and Engbert, 2010), supporting the view that the importance of microsaccades is downplayed in natural unconstrained vision relative to fixation tasks (Collewijn and Kowler, 2008). The peak in scaling exponent seen in all subjects near Δ*t* = 100 ms may be due to the fact that after 3–5 video frames fixation targets have moved far enough to yield drifts larger than expected for a stationary target, or alternatively due to delayed oculomotor feedback with this characteristic time scale (Mergenthaler and Engbert, 2007); additional work is needed to disambiguate these possibilities. Involvement of the latter mechanism would also suggest a means for inducing history effects without a needing precise memory of past gaze positions.

The fact that our observed superdiffusive behavior agrees with that seen in static fixation tasks (Engbert and Kliegl, 2004; Mergenthaler and Engbert, 2007) also shows that increased scaling exponents are simply not due to the pursuit trajectories in our data. Although the peak in turn angle distributions at 0° is partly due to pursuits, this anisotropy alone does not explain the scaling behavior: surrogate walks with the same anisotropy do not yield strong superdiffusivity. It would be ideal to cleanly distinguish “true” fixations and “true” pursuits, but the distinction is highly non-trivial for low target velocities. Our findings suggest that even despite the natural variability of target motion, the overall statistics agree well with those found in typical fixation tasks.

Our use of a head-mounted eye tracker was motivated by the greatly improved subject comfort levels and naturalistic conditions it allows relative to fixed-head systems. This enabled lengthy continuous acquisitions, ideally suited to studying eye movements in film viewing. While slow calibration drifts can hinder head-mounted systems, these are unlikely to affect eye movements occurring within the few hundred milliseconds of each fixation. That is, we focused on gaze trajectories measured relative to fixation onset, not absolute fixation positions, thus requiring high precision rather than high accuracy. Note that the EyeLink II system tracks eye movements in the moving reference frame of the head, and tracks head position in three-space at 500 Hz, compensating for head rotations and translations. Together these measurements yield eye position in the fixed plane of the screen, thus the drifts remain even after accounting for postural sway and vestibulo-ocular corrections. Although postural sway trajectories also exhibit a transition from persistent to antipersistent correlations (Collins and De Luca, 1994), this occurs on much longer time scales than in FEMs (Engbert and Kliegl, 2004), further supporting an origin in the visual system.

We showed that the potential confound of cross-talk between pupil size and position following sudden luminance changes (Kimmel et al., 2012) is not a significant factor for films with relatively slowly-varying luminance. The low noise level of video-based eye trackers (0.01° RMS) is advantageous for studies of small-amplitude FEMs, and the same model of eye tracker has been used to study microsaccades in a head-unrestrained setup (Martinez-Conde et al., 2006). Moreover, the spectral power of the machine noise has been shown using an artificial pupil to be significantly smaller than that observed for a real eye (Wallis, 2006), and the near-flat spectrum corresponds to a near-uncorrelated noise source which is thus unable to induce the observed correlation structure. Thus our results are robust against the noise in our video-based recordings. Ultimately the main benefit of head-mounted video-based eye trackers is that they enable measurements under natural free-viewing conditions unencumbered by the onerous head-stabilization and/or physical interference with the eye required of techniques such as dual-Purkinje image systems and scleral coils. Although such systems are capable of lower noise levels, this is associated with such intrusions that limit ecological validity. We hope that drift scaling properties will be measured using these alternative techniques, perhaps shedding light on whether any discrepancies are due to having a contact lens in the eye and using bite-bar stabilization, versus the limitations of video-based pupil tracking, or varying levels of fatigue, for example.

The model developed here is a suitable generative model for use in inversion schemes to estimate parameters from data. Our simple inversion method is a proof of principle for inferring physiological parameters from FEM recordings, and could provide initial estimates for more sophisticated algorithms (e.g., variational Bayes). Indeed with a larger cohort and improved fitting methods we expect that the parameter estimates could be constrained more tightly than the present somewhat-broad ranges, yielding a sharper probe of the underlying physiology. Our inferred memory lengths in the range 6–32 ms are consistent with typical physiological time constants in premotor neurons of the oculomotor system (Cannon et al., 1983; Aksay et al., 2007), though time constants up to 100 ms have also been implicated (Seung, 1996; Seung et al., 2000). Systematic parameter inference across data sets also opens the possibility of studying group differences between normal and clinical populations.

The current model is particularly simple, but admits several straightforward future extensions that might redress the mismatch at long time lags, at the cost of additional parameters. Currently, the memory's influence on the dynamics begins immediately and ends abruptly after a fixed duration. These properties can be modified by turning the memory on only after a delay (to incorporate a latent period before the history is encoded), or for its influence to decay smoothly by setting α = α(*t*) with some characteristic decay rate. Such behavior could also be modeled by setting σ = σ(*t*) to be *increasing* in time, degrading memory precision through “diffusion” of the stored representation. Correlation of drift with the previous saccade direction could also be modeled by retaining some memory of the previous saccade, rather than beginning anew each fixation.

A recent model of FEMs has also incorporated self-avoidance as the key mechanism driving drifts observed in fixation tasks (Engbert et al., 2011; Engbert, 2012). The model encodes history by treating space as a lattice and recording the number of visits to each site, and the random walk proceeds by choosing the least-visited neighbor at each step. The model also includes a confining potential to keep the random walk near the origin, which is needed for the long-time subdiffusive nature of fixation tasks, as well as a mechanism for triggering microsaccades when occupying highly-visited sites. These additional mechanisms are compatible with our model, although we have not included them for simplicity in the absence of compelling evidence for them in our data. For example, *n*(**x**) could be augmented with a radially-varying component to prevent long fixations from straying too far from the target. However, for natural vision, a static confining potential should ideally be modified to account for fixation of moving targets, requiring a mechanism for predicting target trajectories.

The self-avoiding mechanism is a natural way of avoiding adaptation. A feedback system that tends to avoid previously-encoded scene representations (e.g., involving V1) will tend to yield novel transient stimuli and thus stronger neural responses than for a static rapidly-adapted representation. Such behavior improves sampling of fine spatial detail (Donner and Hemilä, 2007; Otero-Millan et al., 2008); indeed even uncorrelated perturbations to retinal images reduce redundancy and enhance feature extraction by removing low spatial frequencies (Kuang et al., 2012). Dependence of drift trajectories on scene statistics might be expected if FEMs act as an optimal search process. Superdiffusive behavior is associated with optimal search strategies (Viswanathan et al., 1999; Sims et al., 2008), and has been argued to explain saccadic scan paths (Brockmann and Geisel, 2000). Memory of past states might alternatively be embodied independently of the visual scene at the level of brainstem premotor neurons (Seung, 1996; Seung et al., 2000), or by motor neurons in the superior colliculus (Mergenthaler and Engbert, 2007; Hafed et al., 2009; Engbert et al., 2011), or in cortical areas (Pierrot-Deseilligny et al., 2004). All mechanisms that involve a memory of eye position must overcome challenges of biological noise. We argue that models will ultimately need to encode only imprecise history representations, similar to our implementation here. Integrated with brain imaging, the present approach has the potential to greatly inform the neural mechanisms underlying the accumulation of evidence about the statistical structure of natural scenes (Levy et al., 2004) and the temporal hierarchies of human narratives (Hasson et al., 2008; Honey et al., 2012).

### Conflict of interest statement

The authors declare that the research was conducted in the absence of any commercial or financial relationships that could be construed as a potential conflict of interest.
